# Injury-related fear in athletes returning to sports after anterior cruciate ligament reconstruction - A quantitative content analysis of an open-ended questionnaire

**DOI:** 10.1016/j.asmart.2021.03.001

**Published:** 2021-04-07

**Authors:** Shunsuke Ohji, Junya Aizawa, Kenji Hirohata, Takehiro Ohmi, Sho Mitomo, Hideyuki Koga, Kazuyoshi Yagishita

**Affiliations:** a Clinical Center for Sports Medicine and Sports Dentistry, Tokyo Medical and Dental University, 1-5-45 Yushima, Bunkyo-ku, Tokyo, 113-8519, Japan; b Department of Physical Therapy, Juntendo University, 3-2-12 Hongo, Bunkyo-ku, Tokyo, 113-0033, Japan; c Department of Joint Surgery and Sports Medicine, Tokyo Medical and Dental University, 1-5-45 Yushima, Bunkyo-ku, Tokyo, 113-8519, Japan

**Keywords:** Anterior cruciate ligament repair, Fear of re-injury, Fear of movement, Return to sports, Quantitative content analysis

## Abstract

**Background/objective:**

Injury-related fear during sport activities are major psychological factors inhibiting a person’s return to sports (RTS) following anterior cruciate ligament reconstruction (ACLR). Currently, there are no studies that quantitatively analyse the open-ended questionnaire for knee injury-related fear in post-ACLR athletes.

The purpose of this study was to identify knee injury-related fear in athletes who return to ball-centric sports via the use of an open-ended questionnaire. We aimed to determine the main concepts of injury-related fear according to sex, type of sport, and participation level.

**Methods:**

In this study, a quantitative analysis of an open-ended questionnaire was used to examine the type of fear athletes experience after returning to ball-centric sports. The RTS and fear questionnaire collected open-ended questionnaire to knee injury-related fear during sport activities; this questionnaire was completed at the outpatient visit post-RTS. Quantitative content analysis was performed to extract frequently occurring words from the responses to the questionnaire to create a co-occurrence network. The resulting co-occurrence network and extracted words were used to create concepts regarding knee injury-related fear. The relationship between each concept and subject demographics (sex, returned sports events, and participation level) were analysed using the chi-squared test.

**Results:**

Fifty-four athletes (30 females and 24 males) aged 16–45 [median age: 21.2; interquartile range (IQR): 11.0] years with an average RTS of 8.0 (IQR: 3.3) months from ACLR participated in the study. A total of 79 responses were included in the analysis. The knee injury-related fear can be summarized as follows: (1) Quick response to the opponent; (2) Ball-related play; (3) Jump-landing; (4) Contact; (5) Loss of balance; and (6) Athletic movement. Chi-squared tests showed that athletes participating in sport events with potential contact with an opponent (soccer, futsal, basketball, handball, lacrosse, and ultimate (frisbee)) were more likely to experience fear in quick response to the opponent (P < 0.01, adjusted residual = 2.943, ϕ = 0.301).

**Conclusion:**

The knee injury-related fear can be summarized into six concepts. Post-ACLR athletes participating in ball-centric sports need to assess fear in situations such as quick responses to the opponent’s movements and ball-related play, in addition to simple movements such as jumping, cutting, and contact.

## Introduction

Anterior cruciate ligament (ACL) injury is a typical injury in athletes who participate in ball-centric sports involving a sudden change of direction, jump-landing, and contact.^1^^,^^2^ Most athletes with ACL injuries undergo ACL reconstruction (ACLR) with the hope of a return to sports (RTS) at the same level of competition as before the injury.^3^ However, in a meta-analysis examining the rate of RTS for post-ACLR athletes, only 63% of athletes achieved RTS at the same level of competition before the injury.^4^

Post-ACLR athletes are more likely to experience fear of movement and re-injury during sport activities. These fears are a major psychological factor inhibiting RTS following ACLR.^5^ Several studies have shown that athletes who have not been able to RTS at the same level of performance after ACLR have a greater fear than those who have been able to RTS.^6^, ^7^, ^8^

In a survey of athletes RTS after ACLR, Meierbachtol et al. showed that cutting, contact, and jumping were the movements they feared during sporting activities.^9^ These situations and movements are consistent with the occurrence of the ACL injury.^1^ Thus, post-ACLR athletes’ fear situations and movements are similar to those of the injury, even after RTS.

Meierbachtol et al. analysed the fearful movements during sporting activities of post-ACLR athletes without limiting the participants’ sports events.^9^ Here, most of the participants responded to closed-ended questions even though they were instructed to ‘feel free to write in other tasks’. The knee injury-related fear may vary depending on the sport in which they participate. While closed-ended questionnaires are easy to answer and are excellent for quantitative analysis, they do not capture additional data beyond what is selected. An open-ended questionnaire allows us to obtain specific details about the knee injury-related fear. Therefore, analysing the responses to the open-ended questionnaires may reveal the potential fears of post-ACLR athletes. To the best of our knowledge, there are no current studies that have quantitatively analysed the open-ended questionnaires for knee injury-related fear in post-ACLR athletes who participate in ball-centric sports.

The purpose of this study was to determine knee injury-related fear during sport activities after ACLR through quantitative content analysis of an open-ended questionnaire. We aimed to delineate individual concepts and identify characteristics of these concepts by subcategories such as sex, type of sport, and participation level.

## Materials and methods

### Participants

Participants who had undergone primary ACLR between April 2013 and November 2019 and completed an RTS questionnaire were included in the study if they met the following criteria: (1) aged between 16 and 45 years at the time of measurement, (2) participation in sports with a modified Tegner Activity Scale^10^ ≥ 5 before ACL injury, and (3) permission to RTS by an orthopaedic surgeon. Exclusion criteria included the following: (1) ACL injury in the contralateral knee or an ACL re-injury to a reconstructed knee, (2) multiple ligament reconstruction, (3) additional surgery before the RTS, and (4) a return to collision sports (i.e. rugby or American football), martial arts (i.e. judo or karate), or snow and ice sports.

### Surgical technique and postoperative rehabilitation

The autograft sources were bone-patellar tendon-bone or semitendinosus. The surgery using semitendinosus was performed with an anatomical double-bundle reconstruction. If semitendinosus alone was insufficient as a graft tendon, gracilis was added. Range of motion and muscle isometric contraction exercises were started 3 days after surgery. A Straight-Position Knee-Joint Immobilizer (Knee brace, ALCARE Co., Ltd., Tokyo, Japan) and crutches were used and then removed gradually 4 weeks after surgery. Jogging started 3 months after surgery, and the running speed was gradually increased. Partial sport participation was allowed when the following was achieved: at least 6 months after surgery; stroke test^11^ was 1+ or less; > 80% running speed compared to that before injury; > 80% limb symmetry index (LSI) of the single-leg hop distance; and sufficient strength recovery, i.e., >80% LSI of extension and flexion, measured with a BIODEX System 4 Isokinetic Dynamometer (BIODEX Medical Inc., Shirley, NY, USA) at 60°/s and 180°/s.^12^

Participants who underwent repair of the middle-posterior segment of the meniscus were prohibited from performing deep squatting to more than 90° until 3 months after surgery.^12^

### Procedures

Health information including demographics, surgical technique, type of sport, pre-injury participation level, and post-operative subjective athletic performance (PoSAP)^12^ were collected from the self-reported questionnaires and medical records. The fear questionnaire was administered at the outpatient visit after the participant’s RTS. Ethics approval was obtained from the Ethics Committee of Tokyo Medical and Dental University (approval number: M2019-019). All subjects provided written informed consent prior to participation.

### Self-reported questionnaire

The PoSAP asked the question, ‘What is the subjective performance intensity of the sport you are currently participating in?‘. The PoSAP index ranges from 0% to 100% and reflects the athlete’s performance level relative to his or her pre-ACL injury performance.^12^

The RTS and fear questionnaire (appendix) collected the participant’s sport event, the date of their return, and the knee injury-related fear (up to five). For this study, the definition of RTS included not only full participation in the sport events, but also partial participation and practices.

### Quantitative content analysis

This study used KH coder (Version 3. Alpha 1.7k), a software program developed by K. Higuchi for quantitative content analysis.^13^ Quantitative content analysis consists of two steps. The first step analyses text data without subjectivity or prediction of the analyst through automatic processing of the text. The second step creates concepts from the text based on the analyst’s point of view and analyses them statistically.^13^

### First step (analysis of text data)

A verbatim record was made using Excel (Microsoft Excel 2016; Microsoft Corporation, Redmond, WA) of the knee injury-related fear obtained from the questionnaire. The terms ‘right knee’ and ‘left knee’ were changed to ‘operative side’ and ‘non-operative side’, respectively, for each participant.

Morphological analysis was used to divide the text into word units and identify the parts of speech for each word. The frequency of the extracted words was also calculated. In the morphological element analysis, ‘single’ and ‘leg’ were assigned to ‘single-leg’, and ‘change’ and ‘direction’ were assigned to ‘change of direction’. Subsequently, the words commonly used in the same sentence (co-occurrence) were analysed using a co-occurrence network.

### Second step (creating the concept and statistical analysis)

A co-occurrence network analysis and key word in context (KWIC) concordance were used to generate concepts (hypothesis codes) for knee injury-related fear. The relationship between the categories of the subjects (sex, returned sports events, and participation level) and each concept was analysed by the chi-square test. The participation levels were categorized as recreational, competitive, and elite.^6^ Participants were categorized based on previous studies^14^, ^15^, ^16^, ^17^ of injury mechanisms as follows: sports events with potential contact with an opponent (soccer, futsal, basketball, handball, lacrosse, and ultimate (frisbee)) or no potential for contact (volleyball, tennis, badminton). If a significant association was found, the effect size (Cramer’s V or ϕ) was calculated. Adjusted residuals were calculated using statistical package for the social sciences ver. 21.0 (IBM Corp, Armonk, NY, USA).

## Results

**Fig. 1:**
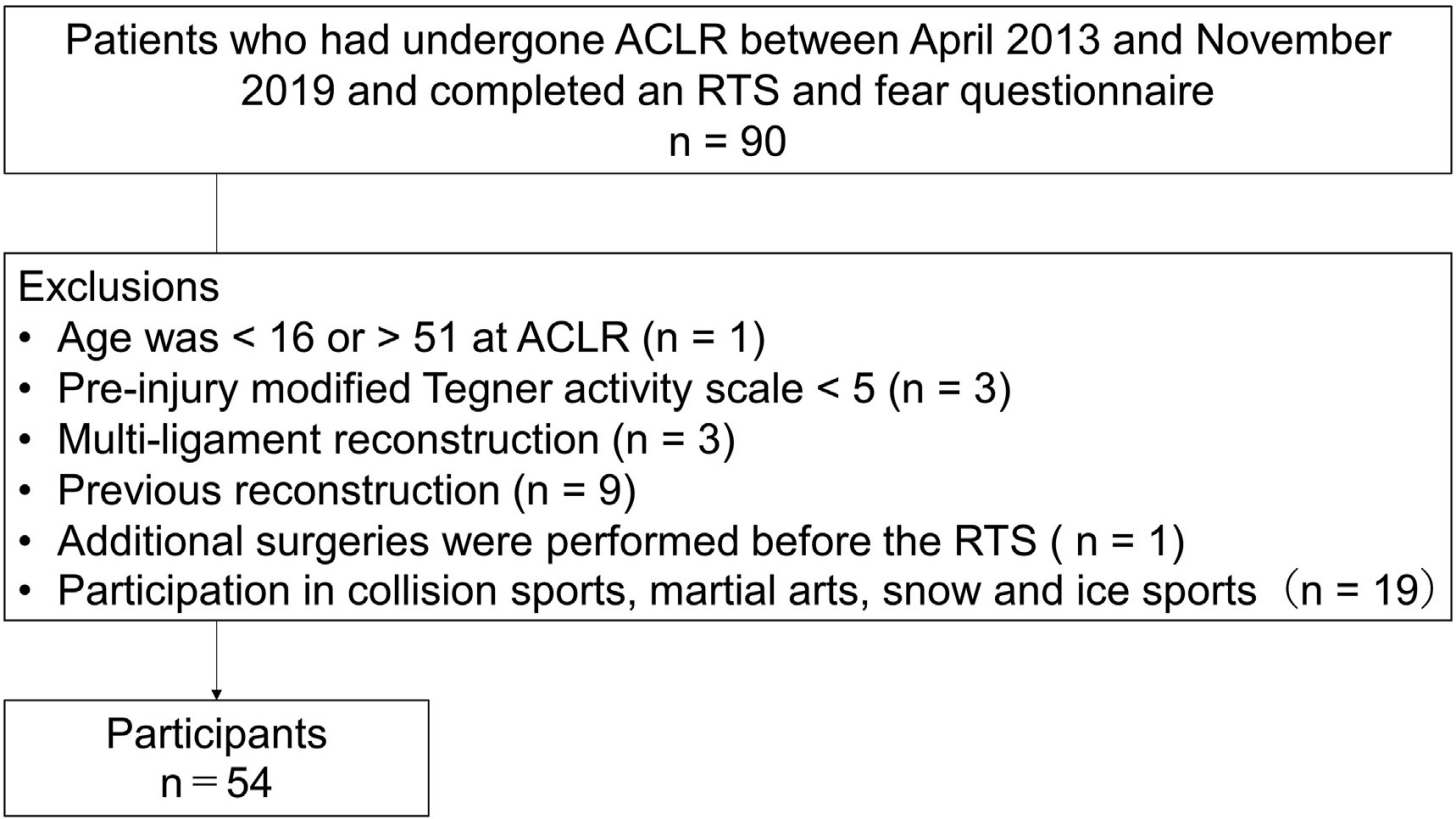
Participants’ flow chart. ACLR, anterior cruciate ligament reconstruction: RTS, return to sports.

Ninety patients responded to the RTS and fear questionnaire, of which 54 met the criteria. There were 30 females and 24 males with a median age of 21.2 [interquartile range (IQR): 11.0] years; pre-surgery modified Tegner activity scale 7.0 (IQR: 4.0); PoSAP, 70.0 (IQR: 20.0)%; and months from ACLR to RTS 8.0 (IQR: 3.3) months. These 54 individuals were eligible for analysis in this study (Fig. 1). All autografts were taken from the semitendinosus. Thirty-five participants underwent meniscus repair. The participants’ sport events were as follows: soccer, 15; basketball, 10; volleyball, 8; badminton, 6; futsal, 5; tennis, 5; lacrosse, 3; handball, 1; ultimate (frisbee), 1.

Seventeen participants responded to the RTS and fear questionnaire but omitted the question about the knee injury-related fear. A total of 79 responses were included in the analysis. The most frequent words were ‘jump’, ‘opponent’ and ‘landing’, followed by ‘defence’, ‘ball’, ‘quick’, ‘cutting’, ‘contact’, and ‘motion’ (Table 1).

**Table 1:** Frequency of extracted words of an open-ended questionnaire.

Extracted words	Frequency
jump	12
opponent	11
landing	11
defence	8
ball	6
quick	6
cutting	5
contact	5
motion	5
stop	4
dash	4
block	4
knee	4
kick	3
shot	3
play	3
heading	3
foot	3
chase	3
single-leg	3
change of direction	3
side	2
step	2
drive	2
balance	2
rebound	2
fear	2
catch	2
full	2
correspondence	2
against person	2
fall	2
hold	2
movement	2
loss	2
direction	2

**Fig. 2:**
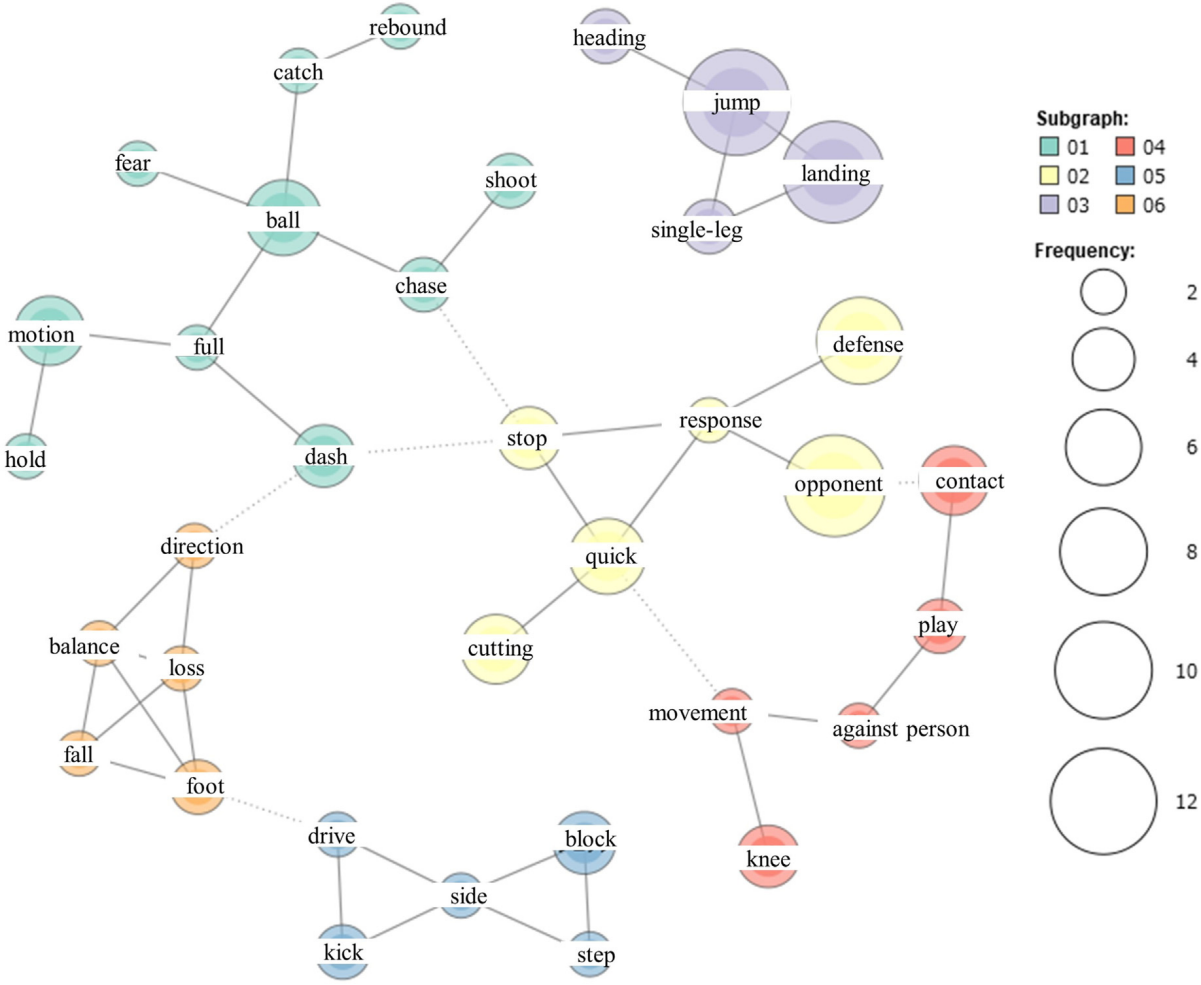
Co-occurrence network. Frequently co-occurring terms in the network are connected by lines (edges), the relative frequency of terms is indicated by the relative size of their node (circle), and the relative frequency of co-occurrence of terms is indicated by the relative thickness of the lines connecting their nodes.

The co-occurrence network of extracted words is shown in Fig. 2, where KWIC concordance confirmed that the extracted words were not conflicting. This resulted in the following six concepts (hypothesis codes) of knee injury-related fear: (1) Quick response to the opponent; (2) Ball-related play; (3) Jump-landing; (4) Contact; (5) Loss of balance; and (6) Athletic movement. Table 2 shows a breakdown of each concept: quick response to the opponent, ball-related play, and jump-landing accounted for 31.7%, 24.1%, and 21.4%, respectively.

**Table 2:** Concepts of knee injury-related fear generated from co-occurrence networks and key words in context concordance.

Concepts	Words	Frequency (%)
Quick response to the opponent	defence	25 (31.7%)
	opponent	
	stop	
	quick	
	cutting	
	response	
	stop	
	hold	
Ball-related play	ball	19 (24.1%)
	chase	
	catch	
	shoot	
	rebound	
	dash	
	motion	
	hold	
Jump-landing	jump	17 (21.4%)
	landing	
	single-leg	
	heading	
Contact	against person	11 (13.9%)
	contact	
	knee	
	play	
	movement	
Athletic movement	block	8 (10.1%)
	side	
	step	
	drive	
	kick	
Loss of balance	balance	6 (7.6%)
	fall	
	loss	
	direction	
	foot	

There were 11 (13.9%) responses that did not correspond to the concept.

Frequency includes duplicate responses, so the total exceeds 100%.

There was no significant association between sex and the participation level in returned sports events (Table 3, Table 4, Table 5). There was a significant association between quick response to the opponent (P < 0.01). The residual analysis revealed that significantly more participants in events with potential contact were fearful of quick response to the opponent (adjusted residual = 2.943, ϕ = 0.301) (Table 4).

**Table 3:** Relationship between six concepts and sex.

Six concepts of knee injury-related fear		Sex		Chi-square value
		Female	Male	
Quick response to the opponent	Yes	13	12	0.269
	No	33	21	
Ball-related play	Yes	12	7	0.054
	No	34	26	
Jump-landing	Yes	11	6	0.111
	No	35	27	
Contact	Yes	5	6	0.356
	No	41	27	
Athletic movement	Yes	5	3	0.000
	No	41	30	
Loss of balance	Yes	4	2	0.000
	No	42	31	

∗P < 0.05, ∗∗P < 0.01.

“Yes” indicates that the participant has a fear that corresponds to each concept.

**Table 4:** Relationship between six concepts and sports events (possibility of contact with the opponent).

Six concepts of knee injury-related fear		Possibility of contact with the opponent		Chi-square value
		Yes	No	
Quick response to the opponent	Yes	23	2	7.182∗∗
	No	32	22	
Ball-related play	Yes	14	5	0.024
	No	41	19	
Jump-landing	Yes	10	7	0.632
	No	45	17	
Contact	Yes	9	2	0.354
	No	46	22	
Athletic movement	Yes	5	3	0.003
	No	50	21	
Loss of balance	Yes	4	2	0.000
	No	51	22	

∗P < 0.05, ∗∗P < 0.01.

“Yes” in each concept indicates that the participant feels the fear corresponding to each concept.

Potential for contact with an opponent: soccer, futsal, basketball, handball, lacrosse, ultimate (frisbee).

No potential for contact with an opponent: volleyball, tennis, badminton.

**Table 5:** Relationship between six concepts and participation level.

Six concepts of knee injury-related fear		Participation Level			Chi-square value
		Recreational	Competitive	Elite	
Quick response to the opponent	Yes	5	17	3	0.403
	No	14	35	5	
Ball-related play	Yes	3	12	4	3.686
	No	16	40	4	
Jump-landing	Yes	2	13	2	1.790
	No	17	39	6	
Contact	Yes	3	8	0	1.442
	No	16	44	8	
Athletic movement	Yes	1	6	1	0.657
	No	18	46	7	
Loss of balance	Yes	1	5	0	1.107
	No	18	47	8	

∗P < 0.05, ∗∗P < 0.01.

“Yes” indicates that the participant has a fear that corresponds to each concept.

## Discussion

This study used an open-ended questionnaire to analyse the characteristics of knee injury-related fear among athletes who participated in ball-centric sports after ACLR. Based on the results of this study, the knee injury-related fear could be summarized into six concepts. Among them, athletes who participated in sports with potential contact with an opponent (soccer, basketball, handball, lacrosse, frisbee) were more likely to experience fear during a quick response to the opponent. This study is the first quantitative content analysis of knee injury-related fear during sports activities after ACLR.

In a survey among athletes who RTS after ACLR, the data showed that cutting, contact, and jumping were the most common movements they feared during sporting activities.^9^ In this study,^9^ most of the subjects responded to closed-ended questions, although the subjects were instructed to ‘feel free to write in other tasks’. While closed-ended questionnaires are easy to answer and are excellent for quantitative analysis, they do not capture anything other than the selected options. In the present study, a quantitative content analysis of an open-ended questionnaire showed the knee injury-related fear of post-ACLR athletes who participated in ball-centric sports. In addition, the previous study did not specify a sporting event, which resulted in a mixture of characteristics of various types of sporting events. As the present study was limited to ball-centric sports that require sudden deceleration, including jump-landings, and changes of direction, we were able to extract concepts specific to ball-centric sports. The results of the present study provide important data that post-ACLR athletes perceive fear of situations such as response to an opponent and ball-related play in addition to simple movements.

We showed that athletes participating in a ball-centric sports with potential contact with an opponent were more likely to be fearful of quick response to the opponent. This suggests that in addition to corresponding to the movements of the opponent, the possibility of contact with the opponent may increase the knee injury-related fear. For example, in a sport such as badminton, where contact with the opponent is physically restricted by the net, non-contact ACL injuries are more likely to occur during the landing after an attack or smash.^15^ On the other hand, studies analysing ACL injury type showed that contact injury or indirect contact injury rates are more likely to increase in contact events such as soccer, basketball, and handball.^14^^,^^17^ The injuries that are characteristic of these sports may be one of the reasons why athletes participating in sports with potential for contact with an opponent are more likely to be fearful of quick response to the opponent.

Otherwise, quick response to the opponent may relate to a characteristic playing style. In sports such as soccer, basketball and handball, there is a tactical defence called ‘pressing’, which involves adapting to the movements of an opponent. Pressing requires the player to be in a closer situation with the opponent and to respond more quickly to non-anticipatory opponent movements, and ACL injuries are more likely to occur in these situations.^16^ The co-occurrence network (Fig. 2) confirms that quick response to the opponent includes defence movements. This sport-specific playing style may also contribute to knee injury-related fear in quick response to the opponent.

In the present study, except for quick response to the opponent, there were no significant associations by category, such as sex, returned sports events, and participation level. The results of this study suggest that most of these concepts are considered to be common to ACLR athletes who participate in ball-centric sports.

## Clinical implication

All six concepts summarized in this study can be used to guide rehabilitation and training after ACLR. In a clinical setting, clinicians and therapists could evaluate in detail for knee injury-related fear based on these concepts in athletes after ACLR. Particularly in athletes participating in sports with potential for contact with the opponent, training for quick response to the opponent needs to be implemented.

Quantifying the intensity of fear in post-ACLR athletes by using ACL-Return to Sport after Injury (ACL-RSI) scale and/or Tampa Scale for Kinesiophobia is common.^5^^,^^18^ In addition to such quantitative assessments, assessing knee injury-related fear using the six concepts from the present study may be important in planning rehabilitation and training programs for RTS.

## Limitation

This study has several limitations. The exact object of fear was not specifically categorized, i.e., the fear of re-injury or fear of movement, because it was a comprehensive survey of fear of knee injuries. A follow-up study should include assessment of the object of fear in situations and movements. In this study, the characteristics of each sport could not be analysed due to the limited sample size. It will be necessary to increase the sample size and analyse the characteristics by sport in the future. Approximately 30% of the participants answered the RTS and fear questionnaire; however, they did not mention the specifics of injury-related fear. Participants were asked to answer the questionnaire at the outpatient visit after their participation in sports; therefore, the possibility that recall bias occurred cannot be ruled out. It may be necessary to ask participants to fill out the questionnaire immediately after their RTS to avoid variation among participants.

## Conclusion

The present study used quantitative content analysis to characterize the knee injury-related fear in athletes who participated in ball-centric sports after ACLR. The knee injury-related fear could be summarized as (1) Quick response to the opponent; (2) Ball-related play; (3) Jump-landing; (4) Contact; (5) Loss of balance; and (6) Athletic movement. Athletes participating in sports with potential contact with an opponent were more likely to experience fear in quick correspondence to their opponent. The results suggest that post-ACLR athletes participating in ball-centric sports need to assess fear in situations such as quick response to the opponent’s movements in addition to simple movements such as jumping, cutting, and contact.

## Funding

This research did not receive any specific grant from the funding agency in the public, commercial, or not-for-profit sector, and no material support of any kind was received.

## Declaration of competing interest

None declared.
