# Supplementary material for: Injury-related fear in athletes returning to sports after anterior cruciate ligament reconstruction - A quantitative content analysis of an open-ended questionnaire
Source: Asia Pac J Sports Med Arthrosc Rehabil Technol. 2021 Apr 7;25:1–7. doi: 10.1016/j.asmart.2021.03.001 (PMC8058518; doi:10.1016/j.asmart.2021.03.001)
Supplement: Multimedia component 1 [file mmc1.docx]

**Return to sports and fear questionnaire**

Name: @PATIENT NAME　　 ID:　 @PATIENT ID　　　　Measure Date: @SYS DATE

Age: @PATIENT BIRTH　　　　Sex:　@PATIENT SEX

Tegner scale (pre-injury):

operative side: R / L

operative date: 20 / / ACL/LM/MM/MCL/PCL/LCL/Others ( )

Please answer the following questions. Please note that the definition of return to sports is “return to the game (either practice or official) or field of competition prior to the injury”.

- When did you return to your sports?

A.D. / /

- Sports events you returned to.

- Returned level

Field practice / Practice game / Official game

- What is the subjective performance intensity of the sport you are currently participating in? (If the pre-injury level was 100%)

%

- What were some situations or movements that you felt fear about your knee while playing? (Please write it concretely)

1.

2.

3.

4.

5.
